# The role of mentalizing in the relationship between schizotypal personality traits and state signs of psychosis risk captured by cognitive and perceptive basic symptoms

**DOI:** 10.3389/fpsyt.2023.1267656

**Published:** 2023-09-21

**Authors:** George Salaminios, Elodie Sprüngli-Toffel, Chantal Michel, Larisa Morosan, Stephan Eliez, Marco Armando, Eduardo Fonseca-Pedrero, Melodie Derome, Frauke Schultze-Lutter, Martin Debbané

**Affiliations:** ^1^Research Department of Clinical, Educational and Health Psychology, University College London, London, United Kingdom; ^2^Research Department, British Association for Counselling and Psychotherapy, Lutterworth, United Kingdom; ^3^Developmental Clinical Psychology Research Unit, Faculty of Psychology and Educational Sciences, University of Geneva, Geneva, Switzerland; ^4^Developmental Imaging and Psychopathology Lab, Department of Psychiatry, University of Geneva School of Medicine, Geneva, Switzerland; ^5^Department of Psychiatry, Lausanne University Hospital (CHUV), Lausanne, Switzerland; ^6^Department of Psychiatry, University of Geneva, Geneva, Switzerland; ^7^University Hospital of Child and Adolescent Psychiatry and Psychotherapy, University of Bern, Bern, Switzerland; ^8^Department of Educational Sciences, University of La Rioja, La Rioja, Spain; ^9^Translational Research Center, University Hospital of Psychiatry and Psychotherapy, University of Bern, Bern, Switzerland; ^10^Department of Psychiatry and Psychotherapy, Medical Faculty, Heinrich-Heine-University, Düsseldorf, Germany; ^11^Department of Psychology, Faculty of Psychology, Airlangga University, Surabaya, Indonesia

**Keywords:** mentalization, schizotypy, basic symptoms, psychosis, CHR, reflective functioning

## Abstract

**Objective:**

Schizotypal traits and disturbances in mentalizing (the capacity to understand the mental states driving one’s own and others’ behaviors) have been implicated in increased vulnerability for psychosis. Therefore, we explored the associations linking schizotypal traits, mentalizing difficulties and their interactions to clinical high-risk for psychosis (CHR-P), as captured by the Basic Symptoms (BS) approach, during adolescence and young adulthood.

**Methods:**

Eighty-seven adolescents and young adults from the general population (46% male, 44% female; age: 14–23 years) were assessed with the Schizophrenia Proneness Interview (SPI-CY/A) for 11 perceptive and cognitive BS, with the Schizotypal Personality Questionnaire (SPQ) for schizotypal traits, and with the Reflective Functioning Questionnaire (RFQ) for self-reported mentalizing abilities. The RFQ evaluates the level of certainty (RFQc scale) and uncertainty (RFQu scale) with which individuals use mental state information to explain their own and others’ behaviors.

**Results:**

Logistic regression models showed significant positive effects of the SPQ disorganization scale on perceptive BS and of the SPQ interpersonal scale on cognitive BS. Post-hoc analyses revealed that schizotypal features pertaining to odd speech and social anxiety, respectively, were associated with perceptive and cognitive BS. Furthermore, higher scores on the RFQu scale and lower scores on the RFQc scale independently explained the presence of cognitive BS. Finally, significant interaction effects between RFQc and SPQ odd speech on perceptive BS, and between RFQc and SPQ social anxiety on cognitive BS were found.

**Conclusion:**

Our findings suggest that schizotypal traits and mentalizing significantly relate both independently and through their interactions to the presence of cognitive and perceptive BS included in CHR-P criteria. Furthermore, mentalizing dysfunction may contribute in the relation between schizotypal traits and early state signs of CHR-P. Mentalizing may support both detection and early treatment of CHR-P among adolescents and young adults who present with trait risk for psychosis.

## 1. Introduction

Contemporary research suggests that clinical psychosis is a neurodevelopmental disorder that commonly emerges during late adolescence/young adulthood, and is preceded by premorbid and prodromal aberrations manifesting primarily within the perceptual, interpersonal and cognitive domains (1). In doing so, psychosis is expressed along a continuum ranging from relatively stable trait abnormalities, to sub-clinical psychotic manifestations of lesser severity and duration, and finally to the severe reality distortions typically identified in people diagnosed with the clinical form of the illness (2, 3). Importantly, the transition from premorbid and prodromal psychotic manifestations to a clinically diagnosable form of psychosis has been linked to adverse outcomes in social, interpersonal, and occupational functioning that often persist despite symptomatic improvement following psychological or pharmacological treatment (4). For this reason, the focus of clinical intervention is progressively shifting toward a more preventative approach, seeking to identify and treat risk for psychosis during its premorbid and assumed prodromal stages, i.e., the clinical high-risk (CHR-P) stage, prior to the onset of the first clinical episode (5). Several studies suggest that early intervention might improve outcomes and reduce illness-related costs (6, 7). However, the psychological processes that are involved in the earliest stages of psychosis expression and should be targeted early to prevent the onset of clinical illness remain unclear (8).

Two main approaches have been developed for the assessment of the CHR-P stage that most proximally precedes the onset of clinical psychosis: the ultra-high risk (UHR) and the basic symptoms (BS) approaches (9). Although both approaches focus on the detection of newly emergent CHR-P states conferring proximal vulnerability for transition to psychotic disorders, they differ in terms of the manifestations they seek to capture. First, the UHR paradigm primarily relies on the assessment of positive psychotic manifestations (e.g., unusual thought content, persecutory ideas, grandiosity, perceptual abnormalities and disorganized speech) that are too brief or not severe enough for a psychiatric diagnosis of clinical psychosis (10). In contrast, the BS approach relies on the assessment of a wider range of subtle, subjectively experienced shifts in cognitive and perceptual processes, including but not restricted to experiences of thought interference, unstable ideas of reference, attentional problems and language difficulties (11, 12). As such, the assessment of BS has been suggested as a complementary approach that can support the detection of the earliest CHR-P states, prior to the development of attenuated psychotic symptoms (9, 11, 12).

Overall, the UHR and BS approaches have been shown to be sensitive in capturing proximal risk for conversion to clinical psychosis among help-seeking populations, with conversion rates ranging between 15.0% at one year to 29.1% at three years for UHR criteria, and between 25.3% at one year to 50.0% at three years for the BS “cognitive disturbances” (COGDIS) criterion (9). Yet, conversion rates seem to have generally declined in CHR-P samples in recent years (13). At present, the alternative assessment of the two symptomatic UHR criteria based on attenuated and transient psychotic symptoms and COGDIS have been recommended for clinical use (9, 14) to support the timely application of indicated treatments to attenuate the risk for conversion to clinical psychosis (15, 16). Importantly however, beyond the risk of clinical psychosis, CHR-P patients who do not transition to psychotic disorders have been repeatedly reported to have adverse mental health and functional outcomes, including poor social functioning, persistence or development of non-psychotic mental health disorders, and non-remission of CHR-P symptoms (17). Thus, from a clinical perspective, further elucidating the factors, including premorbid vulnerability traits (18), that may potentiate or attenuate psychosis risk will support the application of targeted early prevention treatment approaches aiming to attenuate clinical trajectories at the earliest stages of their expression.

Current approaches to study the first signs of psychosis in non-clinical populations are based on the psychometric evaluation of schizotypal personality traits that capture the phenotypic expression of the underlying genetic liability for schizophrenia-spectrum disorders (19, 20). Contrary to the symptomatic CHR-P states, schizotypal traits are subjectively recognized by individuals as common aspects of their personality functioning (21–23). Most psychometric analyses examining the factorial structure of schizotypal traits typically identify three dimensions: a cognitive-perceptual (positive schizotypy: hallucination and delusion-like phenomena), an interpersonal (negative schizotypy: social anxiety, constricted affect) and a disorganization dimension (odd behaviors and speech) (22, 24). Longitudinal research with study intervals spanning from 5 to 50 years suggests that self-reported schizotypal manifestations represent distal trait markers for the development of psychotic disorders, with heightened negative schizotypy identified as the most consistently reported distal predictor of conversion to psychosis in CHR-P samples (18, 22, 25).

Although schizotypal traits have been related to CHR-P states (23, 26), not all people exhibiting schizotypal trait manifestations develop more dysfunctional or clinically relevant psychotic states (27). Indeed, according to most conceptual models, schizotypal traits are not assumed to be sufficient to indicate risk for clinical psychopathology (22, 28, 29). Rather, state manifestations of psychosis risk, such as those captured by the UHR and BS approaches, may represent clinical exacerbations of schizotypal personality traits; while a second source of risk may emanate from additional neurobiological aberrations (29, 30). This was recently empirically supported (23), yet, the psychological factors that may potentiate the transition from non-clinical schizotypal manifestations to the earliest state signs of psychosis risk, such as self-experienced cognitive and perceptual BS, remain unclear.

An important psychological factor mitigating the development of risk states for psychosis may be mentalizing - the capacity to perceive or interpret one’s own and others’ behaviors, as being driven by intentional mental states, such as thoughts and feelings (31–34). Mentalizing constitutes a multifaceted construct that captures attempts to make sense of oneself and others in terms of subjective mental states. In doing so, mentalizing enables us to form representational models of human behavior in order to adaptively navigate the complexity of the social world, as well as monitor and regulate our own thinking and feeling states (31). Meta-analyses indicate that both schizophrenia and CHR-P patients exhibit dysfunctions in multiple domains of mentalizing (35, 36). More recently, a longitudinal study showed that mentalizing abilities, assessed through the use of narrative-based methodologies, significantly predicted conversion to clinical psychosis in a CHR-P sample (37). Importantly, another line of research indicates that subtle mentalizing difficulties are already present among non-clinical adult and adolescent samples who report schizotypal traits, prior to the development of clinical state manifestations, suggesting an early pathway toward illness expression (38–40). Furthermore, evidence suggest that mentalizing difficulties among adolescents who report schizotypal trait manifestations may contribute to the emergence of clinically-relevant symptoms, including thought problems and delusional ideation (38, 41). Thus, previous findings among both CHR-P and non-clinical samples highlight that mentalizing may play a role in modulating the trajectory of emerging psychosis across the developmental continuum of its expression.

However, to the best of our knowledge, the relationships between schizotypal traits, mentalizing dysfunction, and early state manifestations of psychosis risk, such as those captured by BS criteria, have yet to be simultaneously examined. Elucidating the nature of associations linking schizotypal traits and mentalizing difficulties to the presence of state manifestations relevant for psychosis during the critical developmental window from adolescence to young adulthood may contribute to inform early prevention treatment strategies aiming to attenuate the trajectory of emerging psychosis at its earliest stages.

Thus, the present study seeks to (a) assess the associations of schizotypal traits and self-reported mentalizing with the presence of cognitive and perceptive BS in a sample of community adolescents and young adults, and (b) examine whether schizotypal traits interact with mentalizing difficulties to account for the presence of cognitive and perceptive BS. On the basis of previous research suggesting that schizotypal traits and mentalizing dysfunctions are liked with psychotic symptoms among CHR-P samples (26, 37), we hypothesized that schizotypal traits and self-reported mentalizing difficulties (i.e., high uncertainty and low certainty in mental states) would be independently associated with the presence of both perceptive and cognitive BS. Furthermore, given that mentalizing has been proposed as a psychological factor that may modulate CHR-P among individuals who exhibit premorbid schizotypal trait manifestations (32, 37), we hypothesized that schizotypal traits would account for the presence of cognitive and perceptive BS in the presence of mentalizing difficulties.

## 2. Materials and methods

### 2.1. Participants and procedure

Eighty-seven community adolescents and young adults (47 female, 40 males), aged 14 to 23 years (*M* = 19.27, SD = 2.09) were recruited *via* written advertisements in public schools, universities and community centers in the city of Geneva, Switzerland. None of the participants suffered from past/present psychiatric disorders, or neurodevelopmental disorders (e.g., autism spectrum disorder). Written informed consent was obtained from all participants and legal guardians of those under 18 years of age.

### 2.2. Measures

*The Schizotypal Personality Questionnaire* (SPQ) (42) measures schizotypal traits subjectively experienced as common aspects of one’s personality functioning. It yields three dimensional scores and nine subscale scores: cognitive-perceptual (unusual perceptual experiences, ideas of reference, suspiciousness, odd beliefs or magical thinking), interpersonal (social anxiety, constricted affect, lack of close friends) and disorganization (odd speech, odd behaviors). The French version of the SPQ has shown good reliability (Cronbach’s alpha = 0.91) (42) and has been validated for French-speaking adolescents (43).

Mentalizing was assessed using the French version of the *Reflective Functioning Questionnaire* (RFQ) (44). The RFQ is a brief and easy to administer measure that assesses participants’ self-reported certainty and uncertainty about mental states, reflecting how confident vs. how doubtful one is in utilizing mental state information, such as thoughts and feelings, to explain their own and others’ behaviors. Items are scored on a seven-point Likert scale, ranging from 1 (strongly disagree) to 7 (strongly agree). The *uncertainty about mental states* subscale (RFQu) focuses on the extent to which individuals agree with statements such as ‘Other people’s thoughts are a mystery to me’ and ‘Strong feelings often cloud my thinking’. High scores on the RFQu reflect poor usage of mental state information and a stance characterized by a lack of knowledge about mental states. The *certainty about mental states* subscale (RFQc) focuses on the extent to which individuals disagree with statements such as ‘I do not always know why I do what I do’. RFQc items are recoded so that high scores reflect better usage of mental state information and adaptive levels of certainty about mental states. The RFQ has been shown to correlate with measures of mindfulness, perspective-taking and empathy (45) and its brief nature makes it a suitable assessment tool for the purposes outcome evaluation in the context of clinical settings and clinical trials. The RFQ has been validated for French-speaking adolescents (44) showing satisfactory reliability for both the RFQu (Cronbach’s alpha =0.68) and the RFQc scale (Cronbach’s alpha = 0.74).

*The Schizophrenia Proneness Instrument, Adult version* (SPI-A) (46) for participants aged ≥18 years and the *Child & Youth version* SPI-CY (47) for participants aged <18 years were used to assess the presence of cognitive and perceptive symptoms included in BS criteria that are equal in both versions. COGDIS captures nine cognitive BS (i.e., inability to divide attention, captivation of attention by details of the visual field, thought interference, thought pressure, thought blockages, disturbances of receptive speech, disturbances of expressive speech, disturbances of abstract thinking, unstable ideas of reference). All nine cognitive symptoms from COGDIS were assessed in the current study. In addition to these, COPER also captures two perceptive BS (i.e., visual and acoustic perception disturbances). Because the current study focused on differentiating between cognitive and perceptive symptoms, we also included COPER items that assess the two perceptive symptoms but, for the current recommendation of only COGDIS for clinical use (9), excluded cognitive BS included in COPER only (i.e., thought pressure, derealization, and decreased ability to discriminate between ideas and perception, fantasy and true memories). SPI-A/CY rate cognitive and perceptive BS for the maximum frequency of their occurrence within the past 3 months ranging from 0 (BS has not occurred in the past 3 months) to 6 (BS has occurred daily within the past 3 months). For the purposes of the current study, which only included participants from the general population, BS item scores on the SPI-A/CY were recoded as categorical variables according to their presence or absence: a score of 0 signified the *absence* of the BS (assigned to scores of 0), while a score of 1 signified the *presence* of the BS (assigned to all scores between 1 and 6). According to rating rules, symptoms that were reported as occurring unchanged throughout life (i.e., a score of 7), were also assigned a score of 0 for not representing a risk state manifestation. Finally, sum scores of the two dichotomized perceptive BS scores and the nine dichotomized cognitive BS scores were calculated and again dichotomized (0 = 0 and ≥ 1 = 1) to indicate the presence/absence of any perceptive or cognitive BS.

### 2.3. Statistical analyses

SPSS 23.0 was used for data analyses. Multivariate logistic regressions were performed to assess the effects of SPQ and RFQ scores as well as their interactions on the presence of perceptive and cognitive BS. In order to allow comparisons between variables, SPQ and RFQ continuous scores were transformed into *z* scores. Because participant gender, age, education level and nationality were not significantly associated with the presence of cognitive and perceptive BS in the current sample, these were not entered as covariates in the analyses.

First, two multivariate logistic regression analyses were conducted using the enter method to examine the effects of the three SPQ dimensions on perceptive and cognitive BS. In the first model, all SPQ scales were entered together as independent variables and perceptive BS was entered as the dependent variable, while in the second model, cognitive BS was entered as the dependent variable. Next, post-hoc regression analyses were conducted using the enter method to examine which subscales of each significant SPQ dimension drove the effect on perceptive and cognitive BS.

Second, two multivariate logistic regression analyses were conducted using the enter method to examine the effects of RFQ scales (RFQu and RFQc) on perceptive and cognitive BS. The two RFQ scales were entered as independent variables in each model, while perceptive and cognitive BS scores were entered as dependent variables in each model, respectively.

Finally, multivariate logistic regression analyses using the enter method were conducted in order to uncover possible interaction effects between SPQ subscales and RFQ scales on perceptive and cognitive BS. SPQ subscales shown to have a significant independent effect on perceptive and cognitive BS in the previous analyses, along with RFQ scales were used in the models as predictors.

## 3. Results

### 3.1. Descriptive statistics

Table 1 presents the demographic data of the sample, the frequency of perceptive and cognitive BS, as well as the mean z scores for the SPQ and RFQ scales. While low-frequency BS were common (Table 1), only 1.1% of participants fulfilled COGDIS requirements, and 9.2% COPER requirements for CHR-P.

**Table 1:** Distributions and means of socio-demographic and clinical variables.

Socio-demographic variables	Total (*N* = 87)
*Age (years)*	
Mean (SD)	19.27 (2.09)
Range	14.04–23.73
Sex, *n* (%)	
Female	47 (54%)
Male	40 (46%)
Nationality, *n* (%)	
Swiss	30 (34.5%)
Mixed including Swiss	22 (25.28%)
Other	35 (40.22%)
Parental highest education, *n* (%)	
Lower secondary education (ISCED 0–2)	8 (9.20%)
Upper secondary education (ISCED 3–7)	79 (90.80%)
Participant highest education, *n* (%)	
No information	6 (6.90%)
Lower secondary education (ISCED 0–2)	34 (39,08%)
Upper secondary education (ISCED 3–7)	47 (54.02%)
*Participant occupational status, n (%)*	
Employed	3 (3.45%)
Searching for employment	3 (3.45%)
Still in education	73 (83.91%)
Other	8 (9.19%)
*Clinical variables*	
Any perceptive basic symptoms, *n* (%)	19 (21.80%)
Any cognitive basic symptoms, *n* (%)	31 (35.60%)
SPQ cognitive-perceptual scale, mean *z* score (SD)	−0.04 (6.36)
SPQ interpersonal scale, mean *z* score (SD)	0.15 (4.45)
SPQ disorganized, mean *z* score (SD)	−0.11 (3.45)
RFQu, mean *z* score (SD)	0.09 (3.44)
RFQc, mean *z* score (SD)	−0.22 (4.52)

### 3.2. Simple effects of schizotypal traits and mentalizing on basic symptoms

Regression analyses of the simple effects of SPQ dimensions on the presence of perceptive or cognitive BS showed that the SPQ disorganization dimension had the only significant but weak effect on perceptive BS (*β* = 0.18, *Wald x*^2^ *(1)* = 1.09, *p* < 0.05), while the interpersonal SPQ dimension had the only significant but weak effect on cognitive BS (*β* = 0.11, *Wald x*^2^ *(1)* = 4.52, *p* < 0.05) (Table 2). *Post-hoc* analyses demonstrated that the SPQ odd speech subscale (disorganization dimension) drove the effect on perceptive BS (*β* = 0.24, *Wald x*^2^ *(1)* = 4.47, *p* < 0.05) and that the SPQ social anxiety subscale (interpersonal dimension) drove the effect on cognitive BS (*β* = 0.31, *Wald x*^2^ *(1)* = 9.31, *p* < 0.01) (Table 3). In doing so, effects of the single subscales were slightly larger than the effects of their corresponding dimensions.

**Table 2:** Logistic regression analyses of SPQ scales on perceptive and cognitive BS.

Dependent variables Independent variables	*β*	SE	Wald (df = 1)	*p*	Exp(*β*)	95% CIs of Exp(*β*)
*Perceptive BS*						
SPQ cognitive-perceptual	0.06	0.04	2.27	0.13	1.06	0.98; 1.14
SPQ interpersonal	0.06	0.06	1.09	0.30	1.06	0.95; 1.19
SPQ disorganized	0.18	0.08	5.05	**<0.05**	1.20	1.02; 1.40
*Cognitive BS*						
SPQ cognitive perceptual	−0.03	0.05	0.48	0.49	0.97	0.88; 1.06
SPQ interpersonal	0.11	0.05	4.52	**<0.05**	1.12	1.01; 1.24
SPQ disorganized	0.05	0.08	0.29	0.59	1.05	0.890; 1.23

BS, Basic Symptoms; SPQ, Schizotypal Personality Questionnaire.

**Table 3:** *Post-hoc* regression analyses of SPQ subscales on perceptive and cognitive BS.

Dependent variables	Independent variables	β	SE	Wald (df = 1)	*p*	Exp(β)	95% CIs of Exp(β)
*Perceptive BS*							
	SPQ odd behaviors	0.07	0.15	0.21	0.65	1.07	0.80; 1.44
	SPQ odd speech	0.24	0.12	4.47	**<0.05**	1.28	1.02; 1.60
*Cognitive BS*							
	SPQ social anxiety	0.31	0.10	9.3	**<0.01**	1.37	1.1; 1.68
	SPQ no close friends	−0.16	0.18	0.81	0.37	0.85	0.60; 1.21
	SPQ constricted affect	−0.01	0.22	0.00	0.98	0.10	0.65; 1.53

BS, Basic Symptoms; SPQ, Schizotypal Personality Questionnaire.

Regression analyses of the simple effects of RFQ scales on the presence of perceptive and cognitive BS showed that both RFQc (*β* = −0.12, *Wald x*^2^ *(1)* = 4.51, *p* < 0.05) and RFQu (*β* = 1.76, *Wald x*^2^ *(1)* = 6.14, *p* = 0.01) had statistically significant but again weak effects on cognitive BS (Table 4). No significant independent effects of RFQ scales on perceptive BS were identified.

**Table 4:** Logistic regression analyses of RFQ scales on perceptive and cognitive BS.

Dependent variables Independent variables	*β*	SE	Wald (df = 1)	*p*	Exp(*β*)	95% CIs of Exp(*β*)
*Perceptive BS*						
RFQu	0.09	0.09	1.10	0.30	1.10	0.92; 1.31
RFQc	0.02	0.07	0.07	0.79	1.02	0.88; 1.18
*Cognitive BS*						
RFQu	0.18	0.07	6.14	**<0.05**	1.19	1.04; 1.37
RFQc	−0.12	0.06	4.51	**<0.05**	0.89	0.79; 0.99

BS, Basic Symptoms; RFQu, Reflective Functioning Questionnaire uncertainty about mental states; RFQc, Reflective Functioning Questionnaire certainty about mental states.

### 3.3. Interaction analyses

Because the odd speech and social anxiety SPQ subscales significantly accounted for the presence of perceptive and cognitive BS, respectively, two regression models were computed to examine the interaction effects of (a) SPQ odd speech with RFQ scales on perceptive BS and (b) SPQ social anxiety with RFQ scales on cognitive BS (Table 5).

**Table 5:** Interaction analyses of SPQ subscales and RFQ scales on perceptive and cognitive BS.

Dependent variables	Independent variables	β	SE	Wald (df = 1)	*p*	Exp(β)	95% CIs of Exp(β)
*Perceptive BS*							
	SPQ OddSp	0.23	0.12	3.71	0.05	1.26	1.00; 1.60
	RFQc	0.09	0.09	1.05	0.31	1.09	0.92; 1.30
	RFQu	0.14	0.11	1.67	0.20	1.15	0.93; 1.43
	SPQ OddSp*RFQc	−0.05	0.03	4.28	**<0.05**	0.95	0.90; 1.00
	SPQ OddSp*RFQu	−0.06	0.04	1.82	0.18	0.95	0.87; 1.03
*Cognitive BS*							
	SPQ SocAnx	0.30	0.11	7.46	**<0.05**	1.35	1.09; 1.67
	RFQc	−0.04	0.08	0.20	0.66	0.96	0.82; 1.13
	RFQu	0.07	0.10	0.51	0.48	1.07	0.89; 1.30
	SPQ SocAnx*RFQc	−0.06	0.02	5.34	**<0.05**	0.95	0.90; 0.99
	SPQ SocAnx*RFQu	−0.01	0.05	0.04	0.84	0.99	0.91; 1.08

SPQ OddSp and SocAnx, Schizotypal Personality Questionnaire Odd Speech and Social Anxiety; RFQc and RFQu, Reflective Functioning Questionnaire Certainty and Uncertainty.

**Figure 1:**
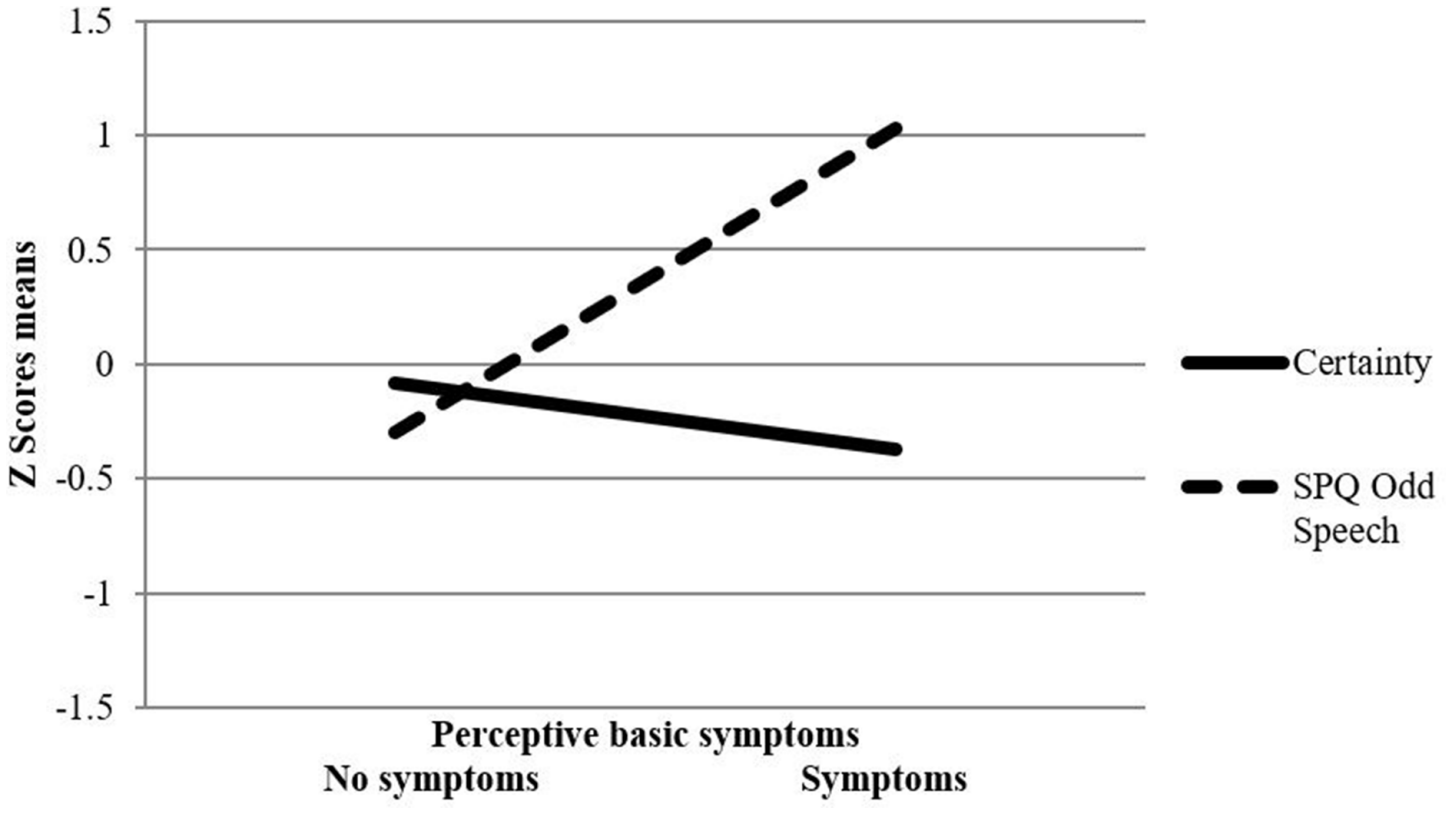
Interaction between SPQ odd speech and RFQ certainty on perspective basic symptoms.

**Figure 2:**
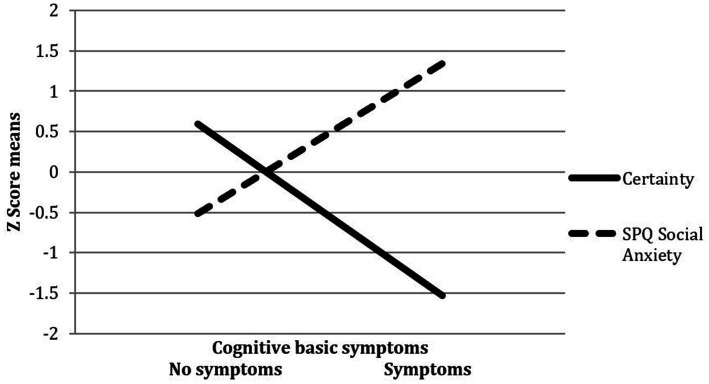
Interaction between SPQ social anxiety and RFQ certainty on basic symptoms.

Regression analyses revealed a significant interaction effect between SPQ odd speech and RFQc on perceptive BS (*β* = −0.53, *Wald x*^2^ *(1)* = 4.28, *p* < 0.05), indicating that the likelihood of experiencing perceptive BS was higher when SPQ odd speech was high and RFQc low (Figure 1). In addition, a significant interaction effect between SPQ social anxiety and RFQc on cognitive BS was found (*β* = −0.57, *Wald x*^2^ *(1)* = 5.34, *p* < 0.05). This revealed that the likelihood of experiencing cognitive BS was higher when SPQ social anxiety was high and RFQc was low (Figure 2).

## 4. Discussion

To the best of our knowledge, this is the first empirical investigation of the associations linking schizotypal traits, self-reported mentalizing and their interactions with the presence of basic symptoms (BS) included in CHR-P criteria. Findings indicate single associations of low effect size of disorganized and interpersonal schizotypal traits with perceptive and cognitive BS, respectively. Furthermore, self-reported mentalizing was independently associated with the presence of cognitive but not perceptive BS. Importantly, schizotypal trait features pertaining to odd speech and social anxiety were shown to interact with self-reported mentalizing difficulties, specifically low certainty in mental states, to account for the presence of perceptive and cognitive BS in our community adolescent and young adult sample. More specifically, results showed that the likelihood of experiencing perceptive BS was higher when odd speech was high and mentalizing certainty was low, while the likelihood of experiencing cognitive BS was higher when social anxiety was high and mentalizing certainty was low.

### 4.1. Effects of schizotypal personality traits and self-reported mentalizing on perceptive and cognitive basic symptoms

The observed a significant association between schizotypal personality traits pertaining to odd speech and perceptive BS is in line with previous studies suggesting that disorganized speech relates to perceptual aberrations in people diagnosed with schizophrenia and non-clinical individuals, and that the two may be underpinned by shared neurobiological substrates (48). Interestingly, two previous studies examining the neurofunctional correlates of schizotypal traits during adolescence in terms of resting-state functional connectivity reported a significant relationship between the disorganized dimension of the SPQ and neural activation in the auditory and visual networks (49, 50). Thus, it might be relevant for future research to investigate the relationship between neural activation patterns, schizotypal traits and perceptive BS among CHR-P groups.

Furthermore, we found a positive association between schizotypal trait features pertaining to social anxiety and cognitive BS. Previous studies have reported higher levels of social phobia among UHR adolescent and young adult samples compared to non-clinical controls, with social phobia in UHR samples being associated to the severity of psychotic symptoms (51). Importantly however, social phobia primarily involves worries about embarrassing oneself due to inadequate behavior, particularly in the eyes of unfamiliar people, while schizotypal social anxiety does not diminish with familiarity and tends to be associated with paranoid fears rather than negative judgments by others (22). Interestingly, findings from a first-episode of psychosis sample suggest that social phobia and paranoid/persecutory ideation may cooccur during the early stages of clinical expression (52). Furthermore, data from a structural equation modeling study showed that although negative schizotypy, mainly represented by social anxiety, was linked to cognitive BS in a CHR-P sample, positive schizotypy had a stronger association (23). Thus, it might be speculated that it is the paranoid/persecutory nature of schizotypal social anxiety that drives the association with cognitive BS. This should be explored in future studies, comparing the association of schizotypal social anxiety and social phobia with cognitive BS. In addition, it might be relevant for future studies to explore whether factors previously shown to underpin social phobia among CHR-P samples, such as self-perceived stigma (53), may also contribute in the relation between schizotypal social anxiety and cognitive BS. Nonetheless, from a clinical point of view, schizotypal social anxiety may constitute an important early prevention target to attenuate the development of CHR-P states.

The association of self-reported mentalizing difficulties (i.e., high uncertainty and low certainty in mental states) with the presence of cognitive BS is in line with a previous studies suggesting that mentalizing difficulties relate to psychosis-relevant thought problems in non-clinical adolescents (38) and to aspects of cognitive disorganization in UHR adolescents and young adults (37). Overall, it appears that mentalizing difficulties are linked to the presence of emerging state manifestations relevant for psychosis risk during adolescence and young adulthood, and may contribute to increased vulnerability for the illness.

Surprisingly, the results of the current study did not show an association of self-reported mentalizing with perceptive BS. This is in contrast to previous studies showing that mentalizing dysfunction relates to perceptual aberrations, such as hallucinations, in clinical psychosis and non-clinical samples (40, 54). It must be noted however, that perceptive BS are clearly distinct from hallucinatory phenomena in that they are not subjectively perceived, at least initially, as external stimuli, but are rather immediately experienced as alterations in one’s own visual and auditory senses, occurring in the perception of real stimuli (46). Thus, it is possible that the association between mentalizing difficulties and perceptual aberrations may only become evident once the latter manifest in the form of attenuated auditory or visual hallucinations. Furthermore, previous studies suggesting associations between mentalizing problems and perceptual aberrations in clinical and non-clinical samples have employed task-based measures of mentalizing, which specifically capture the ability to attribute other peoples’ cognitions. In contrast the RFQ is a self-report measure designed to also capture self-oriented and affect-based aspects of mentalizing. Indeed, previous studies that have either used the RFQ in non-clinical samples or have used narrative-based methodologies to assess mentalizing in UHR samples did not report associations with perceptual abnormalities or hallucinatory phenomena (37, 38). As such, methodological differences in the assessment of both mentalizing and perceptual symptoms may have accounted for the divergence from previous findings.

### 4.2. The role of reflective functioning in the relationship between schizotypal traits and basic symptoms

Results of the current study indicate that schizotypal trait features of social anxiety and odd speech, respectively, interacted with reduced certainty in mental states to account for the presence of cognitive and perceptive BS. In the case of cognitive BS, this interaction presented in addition to the single effect of schizotypal social anxiety, while in case of perceptive BS, only the interaction of mentalizing certainty and odd speech became significant.

The interaction between schizotypal trait features of social anxiety and mentalizing might elucidate some the underlying mechanisms linking schizotypal social anxiety with cognitive BS. In the context of failures to form adaptive representations of one’s own and others’ mental states during interpersonal situations, schizotypal social anxiety may exert a disorganizing effect on cognitive functions (55), which is putatively expressed as self-experienced cognitive BS. Furthermore, cognitive BS might further impair already present mentalizing difficulties, leading to a vicious circle of increasing mentalizing impairment and symptoms. Future studies should employ longitudinal designs to explore whether better mentalizing abilities exert a protective role against the development of cognitive BS among adolescents and young results who present with schizotypal trait features of social anxiety, and whether cognitive BS increase mentalizing difficulties.

Our findings also suggest that the likelihood of reporting the presence of perceptive BS in our sample was higher when odd speech was high and mentalizing certainty was low. Previous studies have shown that disorganization features of schizotypy, including odd speech, are prospectively linked to the developmental trajectory of clinically-relevant perceptual aberrations (56). Our study adds to these findings by suggesting that difficulties in adaptively using mental states to understand one’s own and others’ behaviors may contribute in the experience of perceptual aberrations among individuals presenting with disorganization features of schizotypy.

Overall, the current findings lend support to a model in which mentalizing difficulties contribute in the relation between schizotypal traits and CHR-P relevant symptoms. This resonates with data from Boldrini et al. (37) who found that mentalizing dysfunctions related to symptoms of unusual thought content, persecutory ideas and disorganized communication, as well as increased the likelihood of future transition to a psychotic disorder in a UHR group of young adults. Thus, our results add to those of previous studies suggesting that mentalizing may contribute in modulating psychosis vulnerability across the continuum of its expression, from premorbid trait signs to state manifestations of psychosis risk and toward transition to a first clinical episode. Future studies with large samples may benefit by utilizing network analysis to further elucidate the complex nature of associations linking mentalizing dimensions, schizotypal traits and CHR-P.

### 4.3. Strengths and limitations

While the assessment in a community sample within the age range of highest risk of psychosis and the assessment of BS are clear strengths of our study, some limitations require careful interpretation of the results. First, the data were derived from a relatively small convenience sample and further associations of schizotypal traits and mentalizing with BS could have emerged with a larger more representative sample. In addition, cognitive and perceptive BS in the current study were only analyzed as categorical variables, according to their presence and absence. Thus, we did not examine the relation of schizotypal traits or self-reported mentalizing with the severity of BS, as the latter’s distribution was biased toward low ratings due to the non-clinical nature of the sample. It must also be noted that due to the small sample size and convenience sampling method used, the distributions of certain sociodemographic variables assessed in the current study (i.e., participants’ occupational status and parental education) precluded their inclusion in the analyses as covariates. Similarly, we did not assess social risk factors previously shown to relate to CHR-P, such as childhood trauma (57) and bullying victimization (58), thus, it remains possible that these may have confounded the findings of the study. Furthermore, the cross-sectional nature of the study’s design prevents us from drawing any causal conclusions about the relationships between the variables studied. Finally, on the basis of previous findings from a large representative Swiss community sample with much lower prevalence rates of cognitive and perceptive BS (58), it is likely that our sample has been biased toward people already experiencing some mental health problems and, therefore, presenting with a 2–3 times higher prevalence of BS.

## 5. Conclusion

Despite these limitations, the current study provides preliminary evidence on the relationship between trait and state manifestations relevant for psychosis risk and on the contribution of the psychological process of mentalizing to the relationship between the two. From a clinical standpoint, our results highlight that mentalizing abilities may be a worthwhile target of preventative interventions to sustain resilience against the development of BS and other risk states relevant for psychosis among adolescents and young adults who present with interpersonal and disorganization features of schizotypy.

## Data availability statement

The raw data supporting the conclusions of this article will be made available by the authors, without undue reservation.

## Ethics statement

The studies involving humans were approved by Swissethics (Swiss Association of Research Ethics Committees) project number 2018-00251. The studies were conducted in accordance with the local legislation and institutional requirements. Written informed consent for participation in this study was provided by the participants’ legal guardians/next of kin.

## Author contributions

GS: Conceptualization, Writing – original draft, Writing – review & editing. ES-T: Data curation, Formal analysis, Writing – original draft, Writing – review & editing. CM: Methodology, Writing – review & editing. LM: Data curation, Writing – review & editing, Formal analysis. SE: Conceptualization, Funding acquisition, Resources, Writing – review & editing. MA: Writing – review & editing. EF-P: Writing – review & editing. MD: Writing – review & editing. FS-L: Writing – review & editing, Methodology. DM: Conceptualization, Funding acquisition, Resources, Supervision, Writing – review & editing, Formal analysis, Methodology.

## Funding

The author(s) declare financial support was received for the research, authorship, and/or publication of this article. This work was supported by the Swiss National Science Foundation (100019_159440) and the Gertrude Von Meissner Foundation (ME 7871). The publication of this manuscript was supported by the British Association for Counselling and Psychotherapy.

## Conflict of interest

The authors declare that the research was conducted in the absence of any commercial or financial relationships that could be construed as a potential conflict of interest.

The author(s) declared that they were an editorial board member of Frontiers, at the time of submission. This had no impact on the peer review process and the final decision.

## Publisher’s note

All claims expressed in this article are solely those of the authors and do not necessarily represent those of their affiliated organizations, or those of the publisher, the editors and the reviewers. Any product that may be evaluated in this article, or claim that may be made by its manufacturer, is not guaranteed or endorsed by the publisher.
